# Cold stress resilience of Iranian olive genetic resources: evidence from autochthonous genotypes diversity

**DOI:** 10.3389/fpls.2023.1140270

**Published:** 2023-05-09

**Authors:** Issa Karamatlou, Saeid Navabpour, Khalil Zaynali Nezhad, Roberto Mariotti, Soraya Mousavi, Mehdi Hosseini-Mazinani

**Affiliations:** ^1^ Department of Agricultural Biotechnology, National Institute of Genetic Engineering and Biotechnology (NIGEB), Tehran, Iran; ^2^ Department of Plant Breeding and Biotechnology, Gorgan University of Agricultural Sciences and Natural Resources, Gorgan, Iran; ^3^ Institute of Biosciences and Bioresources, National Research Council, Perugia, Italy

**Keywords:** abiotic stress, climate change, cold stress, genetic diversity, Iranian olive varieties

## Abstract

Olive (*Olea europaea* L.) is one of the most cultivated tree species in Iran. This plant is characterized by its tolerance to drought, salt, and heat stresses while being vulnerable to frost. During the last decade, periods of frost have occurred several times in Golestan Province, in the northeast of Iran, which caused severe damage to olive groves. This study aimed to evaluate and individuate autochthonous Iranian olive varieties with regard to frost tolerance and good agronomic performance. For this purpose, 218 frost-tolerant olive trees were selected from 150,000 adult olive trees (15–25 years old), following the last harsh autumn of 2016. The selected trees were reassessed at different intervals, i.e., 1, 4, and 7 months after the cold stress in field conditions. Using 19 morpho-agronomic traits, 45 individual trees with relatively stable frost-tolerance were reevaluated and selected for this research. Ten highly discriminating microsatellite markers were used for the genetic profiling of the 45 selected olive trees, and, ultimately, five genotypes with the highest tolerance among 45 selected ones were placed in a cold room at freezing temperatures for image analyses of cold damage. The results of morpho-agronomic analyses evidenced no bark splitting or symptoms of leaf drop in the 45 cold-tolerant olives (CTOs). The oil content of the cold-tolerant trees comprised almost 40% of the fruit dry weight, highlighting the potential of these varieties for oil production. Moreover, through molecular characterization, 36 unique molecular profiles were individuated among the 45 analyzed CTOs that were genetically more similar to the Mediterranean olive cultivars than the Iranian ones. The present study demonstrated the high potential of local olive varieties, which would be promising and more suitable than commercial olive varieties, with regard to the establishment of olive groves under cold climate conditions. This could be a valuable genetic resource for future breeding activities to face climate changes.

## 1 Introduction

Plant genetic diversity plays a crucial role in the global economy as it provides the raw materials for different industries such as agriculture, horticulture, forestry, and biotechnology. A rich gene pool can enable some plants to adapt to climate change and maintain biodiversity and sustainable development while providing numerous benefits to human populations. In the recent decade, climate change has become one of the most serious issues in the world. The Middle East region and Iran are also affected by this problem. Spells of extreme heat and cold out of the usual season are among the consequences of climate change (Kaniewski et al., 2023). On the other hand, adaptation enables plants to overcome changes in the environment that can be used as a criterion for selecting plant materials that display high adaptability to new environmental settings (Kawecki and Ebert, 2004; Gibson et al., 2016). Cold stress is a major issue for olive trees, as it can have significant impacts on tree growth, flowering, productivity, and olive oil quality. Olive trees are well adapted to warm Mediterranean climates (Melgar et al., 2009; Ponti et al., 2014; Mousavi et al., 2019; Mousavi et al., 2022) but are susceptible to low temperatures, especially to out-of-season ones and can be damaged irreversibly. Previous studies have shown that low temperatures can result in a range of morphological, physiological, and biochemical changes in olive trees, including changes in the concentration of plant hormones, photosynthesis, and antioxidant defense systems (Gómez-del-Campo and Barranco, 2005; Arias et al., 2015 and Lodolini et al., 2016; Arias et al., 2017; Mougiou et al., 2020). To mitigate the effects of cold stress on olive trees, several strategies have been developed and studied. These include selecting cold-tolerant varieties of olive trees (Karamatlou et al., 2019), using appropriate cultivation practices such as pruning (Sergeeva, 2010), mulch application to protect the roots of the trees, and the provision of frost protection by irrigation or wind machines (Civelek et al., 2017; Dai et al., 2022).

The olive tree (*Olea europaea* L.) is one of the most diverse and cultivated tree species in Iran, occupying about 2.5% of the country’s total fruit orchards. Olive groves in Iran have a total cultivation area of 80,000 ha and an annual production of about 122,000 tons of olive fruits (Ministry of Agriculture Newsletter, 2021). Iran is also known as one of the regions with the greatest diversity of olive germplasm in the Middle East (Mousavi et al., 2017). This genetic heritage is preserved in several olive germplasm banks, including Tarom, Aliabad, and Minudasht collections. The Minudasht olive germplasm bank in Golestan Province includes 21 cold-tolerant olive (CTO) varieties together with 93 unique local varieties that were collected from 19 provinces of Iran, each with different environmental conditions (Hosseini-Mazinani et al., 2013).

In general, when temperatures drop below −7°C, freezing can damage the olive tree at different levels. At −12°C, damages to the aerial parts of the olive tree, major branches, and even the trunk become likely, particularly in November and December, because the trees have not yet been adapted to the low temperatures as an early warning prior to the cold winter (Barranco et al., 2005; Gómez-del-Campo and Barranco, 2005; Karamatlou et al., 2019). Cold acclimation usually occurs through exposure to low temperatures (0°C–5°C) and/or shortened day length, leading to the production of cold-induced proteins (e.g., antifreeze proteins and ice-binding proteins). The production of such proteins in plants plays an important role in inducing plant adaptation to freezing stress (Pedreschi et al., 2010; Arias et al., 2017; Bredow and Walker, 2017). Freezing injury in plant cells results from extracellular ice crystal formation. The freeze/thaw events can also induce xylem embolism in plants and contribute to frost damage and shoot dieback (Schreiber et al., 2013; Charrier et al., 2014). Olive tissues cannot tolerate the formation of ice crystals but are permanently supercooled to prevent ice formation at low temperatures (Arias et al., 2015; Arias et al., 2017).

Frost events in 2008, 2014, and 2016 caused extreme damages to olive trees in the northeast of Iran and inflicted significant economic loss to the olive industry. In general, the frost events in this zone are highly unpredictable in their frequency and occurrence. They can happen both unexpectedly in the late autumn and in the middle of winter (Karamatlou et al., 2018). Symptoms of frost injury usually varies according to the temperature around the canopy of the tree, the duration of the frost, cultivar, tree age, acclimatization, previous crop load, and farming practices, including irrigation and time of pruning (Wisniewski et al., 2003; Gómez-del-Campo and Barranco, 2005; Sergeeva, 2010). During the vegetative growth stage, tissues could be damaged by freezing temperatures. During winter dormancy, however, the same temperatures could have less harmful effects (Turchetti Iturrieta et al., 2014). Shoot tip burn and defoliation, bark split on branches or trunk, limb dieback, and bark and wood discoloration are the most common symptoms of frost damage in olive trees (Denney et al., 1993; Ruiz et al., 2006; Sanzani et al., 2012; Lodolini et al., 2016; Karamatlou et al., 2019; Mougiou et al., 2020; Rodrigues et al., 2022).

Studying cold tolerance in evergreen fruit trees is very complicated (Roy and Basu, 2009), because freezing damage occurs in the field but symptoms become visible at a later date and at different stages of plant growth (Karamatlou et al., 2019). Previous studies have shown that frost tolerance differs significantly among olive cultivars (Denney et al., 1993; Antognozzi et al., 1994; Bartolozzi and Fontanazza, 1999; Barranco et al., 2005; Gómez-del-Campo and Barranco, 2005; Lodolini et al., 2016). Selection for frost tolerance typically involves laboratory/field evaluations of olives under or after frost stress, followed by an evaluation for high-yield potential or stable performance and oil content. Frost tolerance is generally acknowledged in several commercial cultivars, including Ascolana tenera, Leccino, Bouteillan, Picual, and Zard, whereas Coratina, Manzanillo, and Koroneiki are regarded as less tolerant (Sanzani et al., 2012; Lodolini et al., 2016; Rahemi et al., 2016; Karamatlou et al., 2019). Frequent reports have focused on the application of artificial cold treatments that enable screening for cold tolerance in olive cultivars. However, few cases of research have investigated cold-tolerant trees in the natural environment (Denney et al., 1993; Gómez-del-Campo and Barranco, 2005; Lodolini et al., 2016).

The main objective of this study is to find promising CTO trees in multiple stages of natural cold stress that have occurred in the past decade. From Iran, this is the first report that describes healthy CTO trees and compares them with other Iranian and Mediterranean olive varieties.

## 2 Materials and methods

### 2.1 Field conditions and plant materials

**Figure 1:**
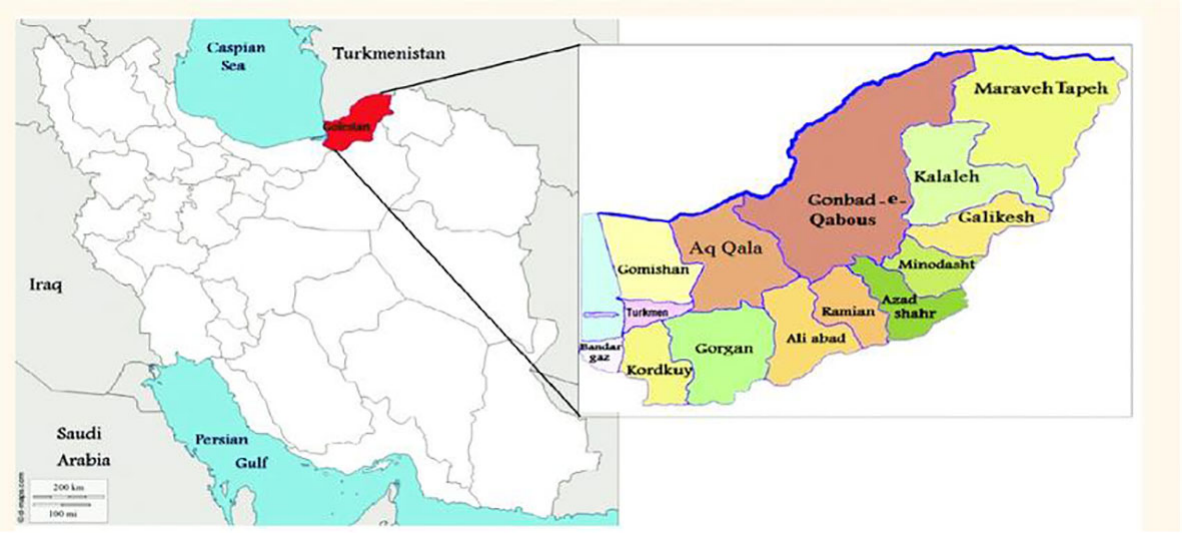
The studied sites in the east of Golestan Province, Iran.

Over the past decade, frost events have occurred several times in Golestan Province, northeast of Iran (**Figure 1**). In this region, olive trees often grow on sloping and rugged land. They are usually planted 7 × 7 m apart, which makes approximately 200 trees ha^−1^. The altitude in this region ranges from 52 to 610 m above sea level. While being mostly rain-fed, the olive orchards are basically comprised of local cultivars.

**Figure 2:**
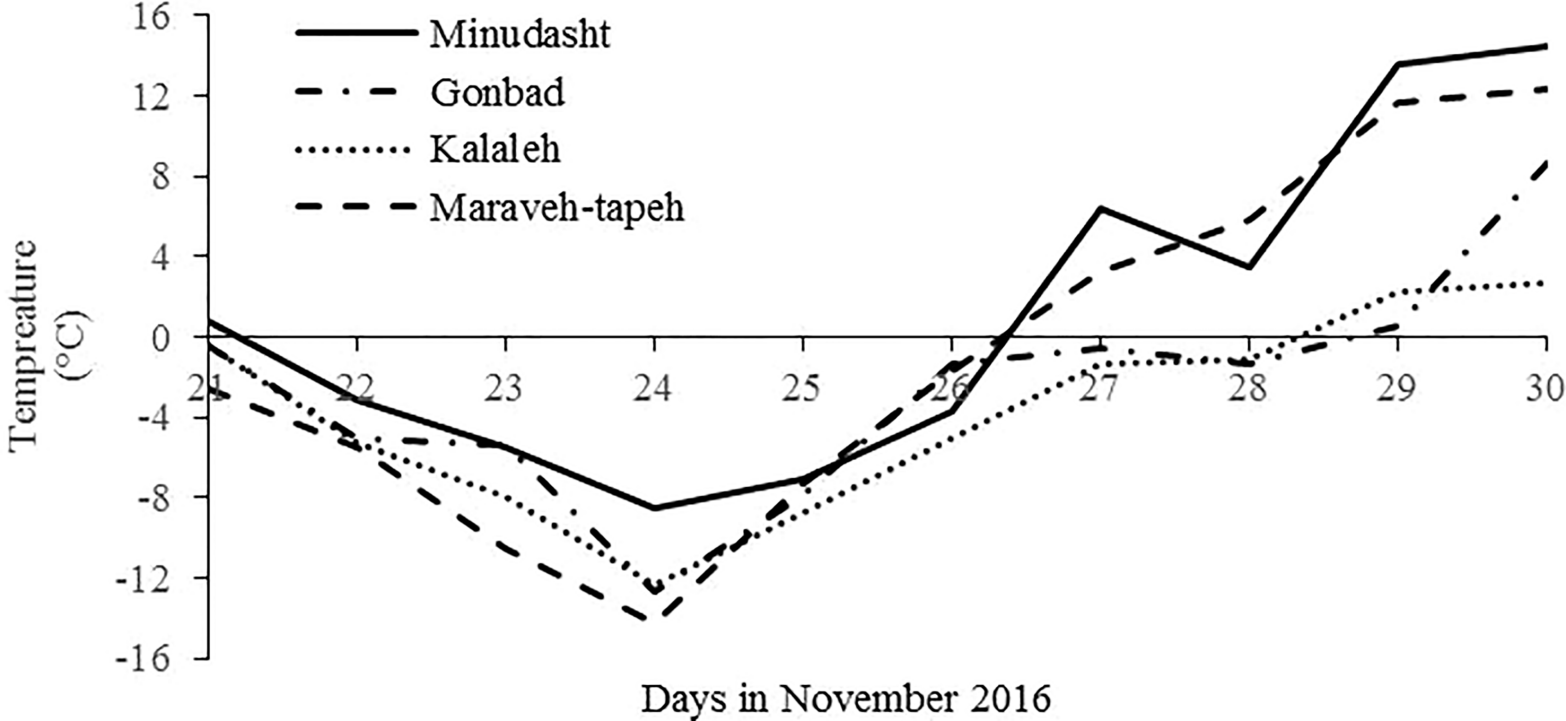
Minimum temperature from 21 to 30 November 2016, in the meteorological stations of the main olive growing areas in the east of Golestan Province.

In 22 to 26 November 2016, an extreme frost occurred in Golestan Province and the temperature suddenly dropped to −8.5°C and −14.2°C in Minudasht and Maraveh-Tappeh regions, respectively (**Figure 2**). The maximum wind speed was 6 m/s in this region at that time. Because it was still in autumn, the olive trees were not acclimated to extreme cold temperatures, and many olive trees were severely damaged. For the correct selection of CTO trees, 10 days after the freezing events, three morphological characteristics were measured, including green and healthy leaves, lack of bark splitting in the branches and trunk, and the absence of changes in bark color. On the basis of the selection criteria and in the first step, 218 tolerant olive trees were selected from 150,000 adult olive trees (aged 15–25) across different olive groves located in eastern Golestan Province. The severity of damage observed on the selected trees was evaluated at intervals of 1, 4, and 7 months after the cold stress. The healthy trees were labeled as CTOs. In the final step and according to the abovementioned selection criteria, 45 local olive trees were selected for further morphological and molecular studies.

### 2.2 Morphological studies

Morphological characterization of CTO was based on Union for the Protection of New Varieties of Plants (UPOV) and International Olive Council (IOC) descriptors, according to a study by Lodolini et al. (2016), which defines 14 morphological characteristics for each tree, three characteristics for the fruit, and two characteristics for the location of the tree (**Table 1**). Because the trees grew under the same agronomic practices, no assessments were made on the effects of horticultural practices, e.g., pruning, nutrition, orchard management, and harvesting time, on the cold tolerance of trees. To determine the direction and percentage of the slopes, a polygon of the olive groves was first drawn using Google Earth software and was then measured using the ArcGIS software. Tree height, canopy height, and average canopy diameter were estimated by taking digital images and analyzing them in the Digimizer software.

**Table 1:** Morphological traits in the 45 selected olive trees, 7 months after the freezing stress.

Row	Trait	Unit	Measurement method
1	Tree age	Year	Year after planting
2	Location altitude	Meter	Google Earth software
3	Dip direction and its percentage	Geographical direction	Cardinal direction
4	Previous yield	Kg	Past average yields
5	Tree height	Meter	Digimizer software
6	Canopy height (H)	Meter	Digimizer software
7	Average of canopy diameter	Meter	Digimizer software
8	Canopy volume	m^3^	CV = 0.5236 × (D)^2^ × H
9	Canopy surface area	m^2^	CSA=3.1416×DH
10	Trunk perimeter	mm	Sewing tape measure
11	Trunk diameter	cm	TD = (TP/2π) × 2
12	Trunk cross section area	cm^2^	TD = π×(TD/2)^2^
13	Length of internode	cm	Ruler
14	Oil content	%	Nuclear magnetic resonance (NMR) spectroscopy
15	Growth habit	Code	1, erect; 2, spreading; 3, drooping
16	Canopy density	Code	1, sparse; 2, medium; 3, dense
17	Canopy defoliation	Code	0, no leaf drop; 1, <50%; 2, >50%; and 3, totally defoliated
18	Bark split ranging	Code	0, none; 1, only on 1-year-old shoots; 2, extended to 2- and 3-year-old branches; 3, extended to primary branches; and 4, extended to the trunk
19	Flowering and fruit set	Code	1, no-flower; 2, flowering and fruit production

### 2.3 Oil content measurement

Three random subsamples (25–30 g) of fresh olive fruits were selected. The number of fruits was counted, and each subsample was weighted independently to calculate fruit weight. Samples were transferred to a forced-air oven at 110°C for 44 h. The subsamples were again weighted to calculate fruit dry weight. The oil content was evaluated using a nuclear magnetic resonance (Bruker Optik GmbH, Ettlingen, Germany), previously calibrated for the range of variation of oil content in different olive cultivars.

### 2.4 DNA extraction and simple sequence repeat (SSR) genotyping

Genomic DNA was extracted from fresh leaves using a plant DNA purification kit according to the manufacturer’s instructions (Exgene Plant SV mini, GeneAll, Seoul, Korea). For genetic analysis, 10 highly polymorphic SSR markers were used, including DCA3-5-9-16-18, EMO90, GAPU71B-101-103A, and UDO-043, which were previously selected as the best-performing loci (Baldoni et al., 2009; Haouane et al., 2011) and were reportedly used in previous occasions of olive genotyping (Mousavi et al., 2017). PCR amplifications were performed in a final volume of 25 µl containing 25 ng of DNA, 10× PCR buffer, 200 μM each dNTPs, 10 pmol of each forward and reverse primer, and 2 U of Q5 High-Fidelity DNA Polymerase (New England Biolabs). All amplifications were performed under the following conditions: 5 min at 95°C, 35 cycles consisting of 25 s at 95°C, 30 s at the appropriate annealing temperature, 25 s at 72°C, and a final elongation at 72°C for 40 min. To distinguish alleles, fluorescent fragments were resolved by capillary electrophoresis in an ABI 3130 Genetic Analyzer (Applied Biosystems-Hitachi) using the internal GeneScanTM-500 LIZ Size Standard (Applied Biosystems). The analyses of samples were performed using GeneMapper genotyping software v3.7 (Applied Biosystems).

To study the relationship between the 45 CTO samples and previously characterized Iranian and Mediterranean varieties, available data from the 10 standard SSR markers were compared with 139 Iranian (Hosseini-Mazinani et al., 2013; Hosseini-Mazinani et al., 2014; Mousavi et al., 2017) and 182 Mediterranean varieties (Trujillo et al., 2014; Mousavi et al., 2017; El Bakkali et al., 2019) reported previously (**Supplementary Table 1**).

For each SSR locus, the number of alleles (Na), effective alleles (Ne), Shannon’s information index (I), observed (Ho) and expected (He) heterozygosity, and fixation index (F) were calculated using GenAlEx 6.5 (Peakall and Smouse, 2012). Polymorphic information content (PIC) was calculated for each microsatellite locus, using CERVUS v.3.0 software (Kalinowski et al., 2007). To determine the genetic relationships of CTO olive accessions with Iranian and Mediterranean varieties, two methods were used: the principal coordinate analysis (PCoA) with GenAlEx 6.5 and the cluster analysis with neighbor-joining (NJ) method that was displayed by Mega7 software (Kumar et al., 2016).

The Bayesian model–based cluster analysis was also used for SSR data through the STRUCTURE v.2.3.4 software (Pritchard et al., 2009) to identify gene pools of the populations. Regarding cluster values, ranging from K = 1 to K = 10, an admixture model and independent allele frequency model were used for a Markov chain Monte Carlo simulation algorithm (MCMC), although no prior information was used for defining the clusters. The length of the burn-in period was set to 100,000, whereas the MCMC after the burn-in period was set to 100,000, and, for each K value, the calculation was repeated 20 times. A method used by Evanno et al. (2005) determined the optimal K value. The program Structure Harvester v.0.9.94 website was used for calculating the optimal value of K, using the deltaK criterion (Earl and vonHoldt, 2012).

Paternity was determined for the 45 CTO varieties, using the maximum likelihood–based method described by Kalinowski et al. (2007) and implemented in CERVUS version 3.0.3 (Marshall et al., 1998). Logarithm of the odds (LOD) scores were computed for each CTO to assign the best candidate parent with 95% confidence. Ten thousand offspring were simulated, allowing for selfing and using the following parameters: The number of candidate fathers was the number of total analyzed varieties in this study. The proportions of “typed loci”, “mistyped”, and “minimum typed loci” were always set at 0.95, 0.05, and 9, respectively, with a relaxed value of 85% and a strict level at 95%.

### 2.5 Evaluation of freezing tolerance through image analysis

After obtaining the results of morphological and molecular analyses, leaf samples from five CTO varieties (CTO-1, CTO-08, CTO-12, CTO-14, and CTO-32) were considered to study the effects of artificial cold treatments, together with the Koroneiki cultivar (as the control, a susceptible variety to frost). In January, 20 healthy mature and cold-acclimated leaves were collected from the selected trees at the same position around the canopy and were exposed to temperatures of 0, −3°C, −6°C, −9°C, −12°C, −15°C, −18°C, and −21°C in a controlled temperature freezer, meaning that eight different temperature treatments were applied. Each temperature treatment was reached within 2 h and was then maintained at that temperature for 4 h. The samples were kept in a refrigerator at 4°C for 24 h after each treatment. Digital images of the leaves were then obtained using an optical scanner. The median lethal time (Lt50), i.e., the temperature at which 50% mortality occurred, was determined using HarFA, Harmonic and Fractal Image Analyzer 5.5.31 software, and GraphPad Prism v8.0.2 by comparing the images through fractal analysis. The methods are described in previous publications (Dennis and Dessipris, 1989; Mancuso, 1999; Mancuso et al., 2003; Azzarello et al., 2009). The degree of cold tolerance was expressed as LT50 by fitting the response curve obtained with a logistic sigmoid function:


Y=(a−d)/(1+e^b(X−c))+d

where x is the treatment temperature, b is the slope at the inflection point, whereas c, a, and d determine the asymptotes of the function. The best fit was determined by the least square’s method (Ingram and Buchanan, 1984). The temperature corresponding to the inflection point of the regression curve, involving a 50% change compared to the control and the totally damaged sample, was considered as an indication of cold tolerance and represented the LT50 value.

## 3 Results

### 3.1 Identification of CTO and morphological evaluation

The identification of CTO trees in the field through natural screening usually involves observing the ability of trees to survive and grow in low-temperature conditions. This can be done by screening olive tree populations in an area that is affected by cold temperatures. Specifically, this is made possible by monitoring their survival rate and overall health. Over time, the trees that have optimally withstood the cold can be identified and potentially used for breeding or propagating CTO trees. Ten days after frost stress in the Golestan Province, damage symptoms were evident on the leaves and branches of olive trees. However, undamaged individual trees were also observed in this period. In the first step of assessments, 218 frost-tolerant trees were identified among a total of 150,000 trees. The evaluation of damage symptoms on frost tolerant trees were continued in the first, third, and seventh months after the frost event. In the second step of assessments, all trees were healthy, but, in the third step, some trees showed withered leaves. In the fourth step, some of the trees were completely withered, showing brown, dry leaves on the tree. Finally, 45 undamaged and healthy olive trees were determined as primarily cold-tolerant.

**Figure 3:**
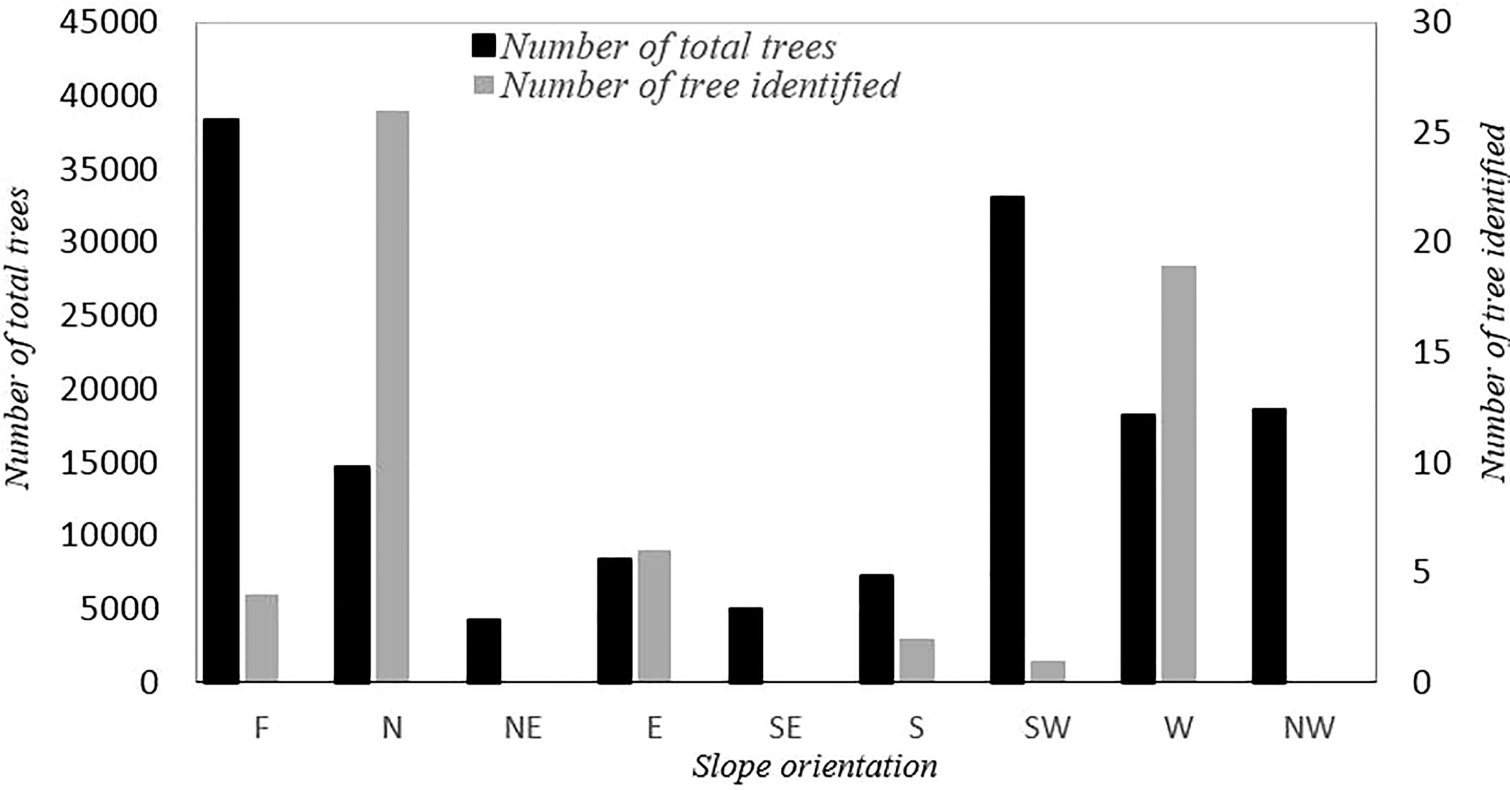
Number of total trees assessed in different geographical directions and number of trees identified in each direction (F, flat; N, north; NE, northeast; E, east; SE, southeast; S, south; SW, southwest; W, west; NW, northwest).

The results of the descriptive statistics for the 45 CTO samples, based on 13 analyzed quantitative variables, are summarized in **Table 2**. The CTOs were located at elevations ranging from 107 to 387 m above sea level, and the slope of the terrain ranged from 0 to 56.8%. The coefficient of variation (CV) of these attributes was 35.08 and 45.89, respectively. The total number of trees assessed at different slopes and the number of CTOs identified are shown in **Figure 3**. There were 14, 16, and six trees of CTOs on the northern, western, and eastern slopes, respectively. In general, 95% of all selected CTOs were in slope-groves, and the remaining (5%) were on flat land. The yield of each CTO tree was estimated on the basis of information obtained from olive growers, ranging from 2 to 60 kg in the same year (data not shown). In the spring of the following year (2017), most of the CTO varieties bloomed, except for 10 trees that failed to bloom. However, because of severe heat stress on May 2017 and with a sudden rise in the temperature to 39.3°C, fruits formed healthily on 24 CTO trees only. The oil content of fruit dry weight showed a wide range of variation from a minimum of 14.95% in CTO-49 to a maximum of 49.91% in CTO-41, with an average value of 39.55%, which showed the high oil potential of the local varieties.

**Table 2:** Statistical descriptive parameters of morphological traits for selecting the 45 olive varieties.

Trait	Maximum	Minimum	Mean	Standard deviation	CV (%)
Tree age (years)	25.00	8.00	16.93	4.85	28.63
Location altitude (m)	411.00	81.00	214.62	84.17	39.22
Percent gradient	56.80	0.00	26.83	13.14	48.99
Yield 2016 (kg)	60.00	0.00	11.50	10.82	94.10
Tree height (m)	6.82	2.00	3.97	1.04	26.27
Canopy height (m)	6.07	1.65	3.40	0.88	25.88
Average of canopy diameter (m)	4.89	1.01	2.68	0.78	29.12
Canopy volume (m^3^)	61.39	1.62	15.10	12.42	82.22
Canopy surface area (m^2^)	83.81	9.65	29.92	15.35	51.29
Trunk diameter (cm)	38.45	8.31	17.13	5.71	33.36
Trunk cross section area (cm^2^)	1,161.25	54.21	255.64	200.35	78.37
Length of internodes (cm)	2.16	1.05	1.55	0.24	15.65
Oil content (%)^*^	49.91	14.95	39.55	8.94	22.60

^*^Oil content in fruits dry weight of 24 CTO which produced fruit (more details are given in **Supplementary Table 2**).

The CV was significantly different regarding all descriptors among the CTO samples, with the highest values observed for the fruit yield and canopy volume traits, whereas the lowest value was attributed to internode length. The analysis of qualitative traits showed that all varieties had medium canopy density and no evidence of bark splitting in 1-year-old shoots. In addition, 97% of the CTO trees had a spreading growth habit (except CTO-9 with an erect growth habit) and finally 86% had no symptoms of leaf drop. Only five varieties showed less than 50% leaf drop.

### 3.2 Molecular diversity of CTO varieties

The genetic diversity of the 45 CTO varieties was estimated using 10 SSR markers (**Supplementary Table 3**). In total, 79 alleles were detected with allele lengths that ranged from 121 to 255 bp. The number of alleles per locus ranged from 5 (DCA18) to 13 (DCA09), with an average number of 7.9 alleles (**Table 3**). The mean number of effective alleles (Ne) was 3.09 and ranged from 1.58 for DCA-05 to 4.67 for DCA-09. The Ho index ranged from 0.31 to 0.97 for the UDO-043 and GAPU-103A loci, respectively, and the He index ranged from 0.36 to 0.78 for the DCA-05 and DCA-09 loci, respectively. The average heterozygosity (0.720) was significantly higher than expected (0.636) and resulted in a negative fixation index (F) for all loci (mean F = −0.110), except in the case of DCA03, DCA-05, DCA-16, and UDO-043, with F values of 0.140, 0.113, 0.109, and 0.258, respectively. PIC ranged from 0.372 (DCA-05) to 0.763 (DCA-09), with an average of 0.602 (**Table 3**).

**Table 3:** The diversity indices of 10 SSR markers detected in 45 CTO olive accessions collected in the east of Golestan Province, Iran.

SSR name	Size (bp)	Na	Ne	I	Ho	He	F	PIC
**DCA-03**	232–255	9	2.677	1.371	0.533	0.626	0.149	0.592
**DCA-05**	194–208	8	1.581	0.872	0.333	0.368	0.093	0.355
**DCA-09**	162–210	13	4.677	1.919	0.956	0.786	–0.215	0.761
**DCA-16**	124–174	8	4.228	1.627	0.644	0.763	0.156	0.727
**DCA-18**	163–187	5	2.456	1.099	0.778	0.593	–0.312	0.526
**EMO-90**	186–198	9	3.204	1.506	0.889	0.688	–0.292	0.655
**GAPU-71B**	121–144	6	3.736	1.485	0.889	0.732	–0.214	0.689
**GAPU-101**	182–218	7	3.894	1.511	0.889	0.743	–0.196	0.700
**GAPU-103A**	136–190	5	2.691	1.149	0.978	0.628	–0.556	0.558
**UDO-043**	172–216	9	1.767	1.040	0.311	0.434	0.283	0.421
**Total**		79	31.25					
**Mean**		7.9	3.091	1.358	0.720	0.636	–0.110	0.598

### 3.3 Genetic relationships, differentiation, and parentage analysis

**Figure 4:**
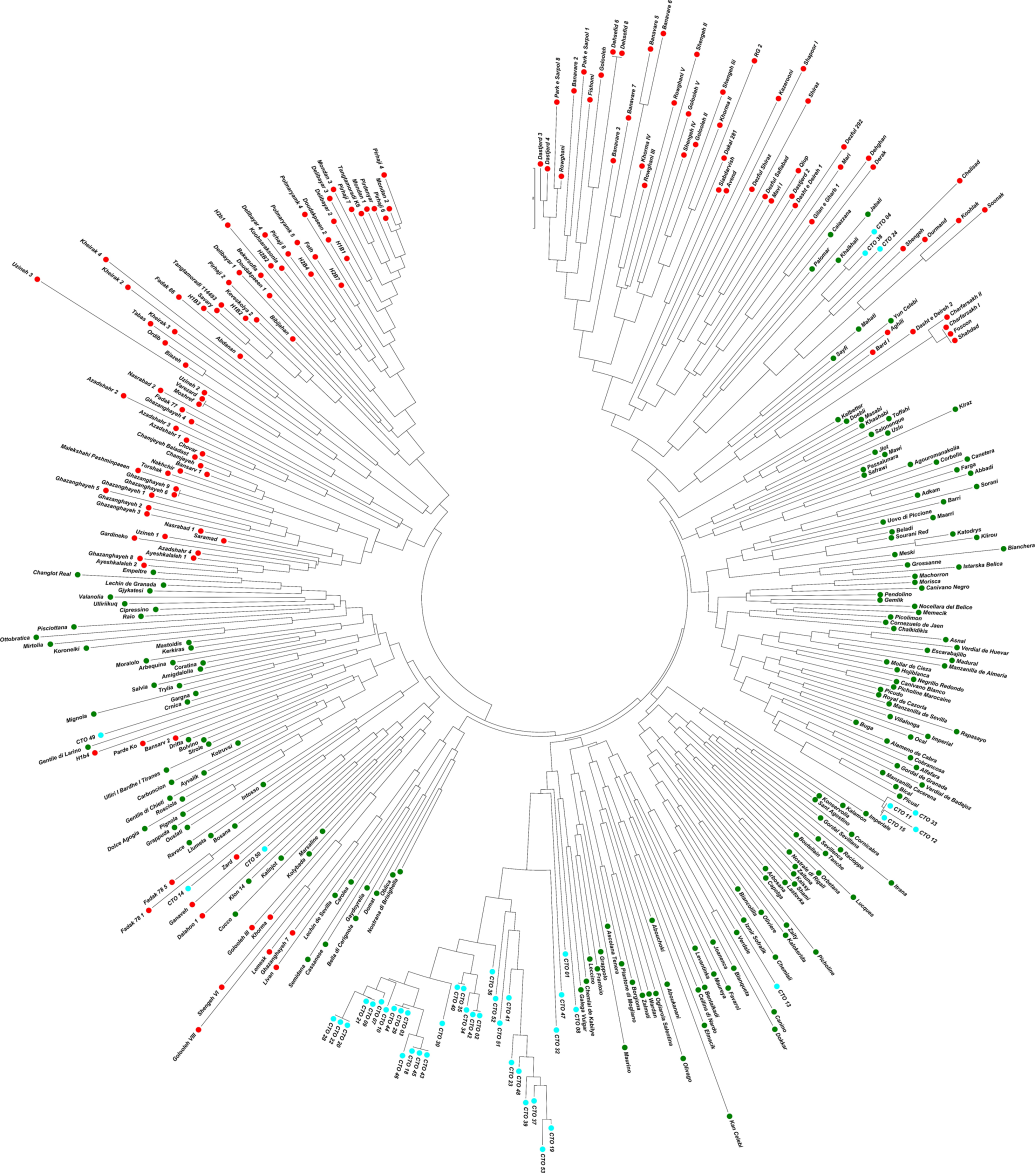
Neighbor-joining dendrogram based on a simple matching dissimilarity matrix created with GenAlex 6.5 and displayed with Mega7 software. It shows the phylogenetic relationships between CTO accessions (light blue) with Iranian olive ecotypes/cultivars (red) and Mediterranean olive cultivars (green) assessed using the SSR markers.

**Figure 5:**
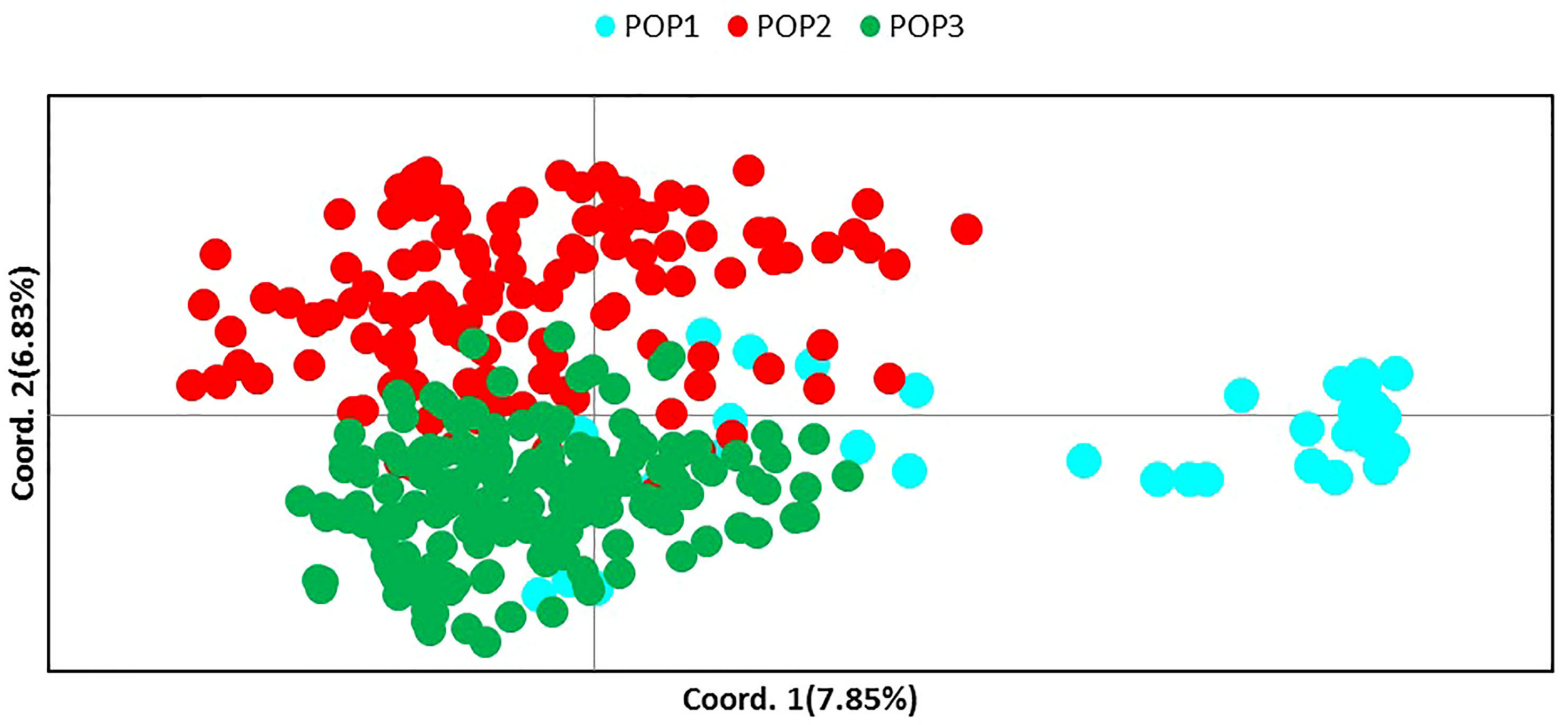
Principal coordinates analysis (PCoA) by GenAlEx 6.5 shows differentiation among CTO (POP1), Iranian olive ecotypes/cultivars (POP2), and Mediterranean olive cultivars (POP3) based on the genetic distance matrix obtained from the variation of SR markers.

**Figure 6:**
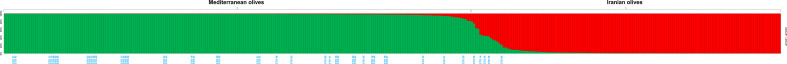
Results of Bayesian model–based clustering method, analyzing CTO trees and a selection of Iranian/Mediterranean olives.

Molecular analysis showed significant variations in the studied CTO samples. To gain additional information about the CTO varieties, the relationship of these samples with 139 Iranian olive ecotypes/cultivars and 182 Mediterranean olive cultivars were studied. A comparison of SSR profiles showed a considerable difference between them and the 45 CTO olive accessions. The results of NJ, PCoA, and population structure showed two main groups after analyzing 366 samples. The first group mostly contained Iranian ecotypes, whereas the second group included all 45 CTO varieties together with all Mediterranean cultivars and the rest of the Iranian ones. Thirty-one CTO varieties were tightly grouped with each other in one subcluster, whereas half of them were placed in groups of two or three identical trees. The rest of the CTO varieties were genetically more similar to the Mediterranean cultivars than to the Iranian olive ecotypes. Within the CTO varieties, six cases of identical genetic profiles were observed. In fact, the CTO-2 was identical to 42, CTO-3 to 29 and 44, CTO-9 to 21, CTO-18 to 43 and 45, CTO-20 to 22 and 28, and, finally, CTO-35 to 40. This finding made it possible to individuate 36 unique genetic profiles that characterized the cold-tolerant olive trees in the present study. CTO-11, CTO-12, CTO-15, and CTO-33 were clustered together with the Mediterranean cv. “Picual”, whereas CTO-50 and CTO-14 were grouped with the Iranian “Zard” cultivar. CTO-13 was clustered together with cvs. “Verdal” from France, “Chemlali” from Tunisia, and “Izmir Sofralik” from Turkey. CTO-4, CTO-38, and CTO-24 were grouped in one cluster, close to the Iranian cultivar “Shengeh”, “Chelisad” ecotype from Khuzestan province, and “Ourmand” and “Soonak” ecotypes from Charmahal province (**Figure 4**). PCoA was performed on a genetic distance-based matrix on a complete dataset of 366 individuals, including 45 CTO varieties, 139 Iranian ecotypes, and 182 Mediterranean cultivars to determine whether partitioning into these groups is supported by genetic variation. The results of PCoA were similar to those of the NJ algorithm (**Figure 5**), and a clear separation of several CTOs was observed while some similarity was shown in the PCoA scatter plot, concerning the analysis of the CTO varieties. Most of the CTO varieties were distinctly different from other varieties, and, only in some cases, also observed in the cluster analysis, they were adjacent to Mediterranean groups. To understand the population structure of the CTO varieties, a Bayesian-based clustering algorithm was performed and the number of most-likely subpopulations (K) peaked at K = 2 (**Figure 6**; **Supplementary Table 4**). All 366 varieties were divided into two main populations. The first population (POP1) included almost all Mediterranean cultivars, whereas the second population (POP2) contained all of the Iranian varieties. The majority of CTO varieties were assigned to POP1, whereas only four CTO varieties were dispersed within the POP2 (**Figure 6**).

Parentage analysis by Cervus on 10 SSR data allowed the identification of direct parentage of 10 of the 45 CTO varieties (**Table 4**). Only two of them belonged to the Iranian ecotype “Ourmand”, whereas all the others were derived from the Mediterranean cultivars, especially “Picual” (40%).

**Table 4:** Parentage analysis by CERVUS, with the lowest mismatch in 45 CTO samples.

Offspring	Loci typed	Candidate parent	Loci mismatching	LOD score
**CTO_04**	10	Ourmand	1	10.41
**CTO_08**	10	Koroneiki	2	3.53
**CTO_11**	10	Picual	1	8.09
**CTO_12**	10	Picual	1	8.0
**CTO_13**	10	Verdale	1	6.99
**CTO_15**	10	Picual	1	8.09
**CTO_33**	10	Picual	1	8.77
**CTO_38**	10	Ourmand	2	5.88
**CTO_49**	10	Frantoio	2	6.31
**CTO_50**	10	Buga	1	2.30

### 3.4 Freezing tolerance

**Figure 7:**
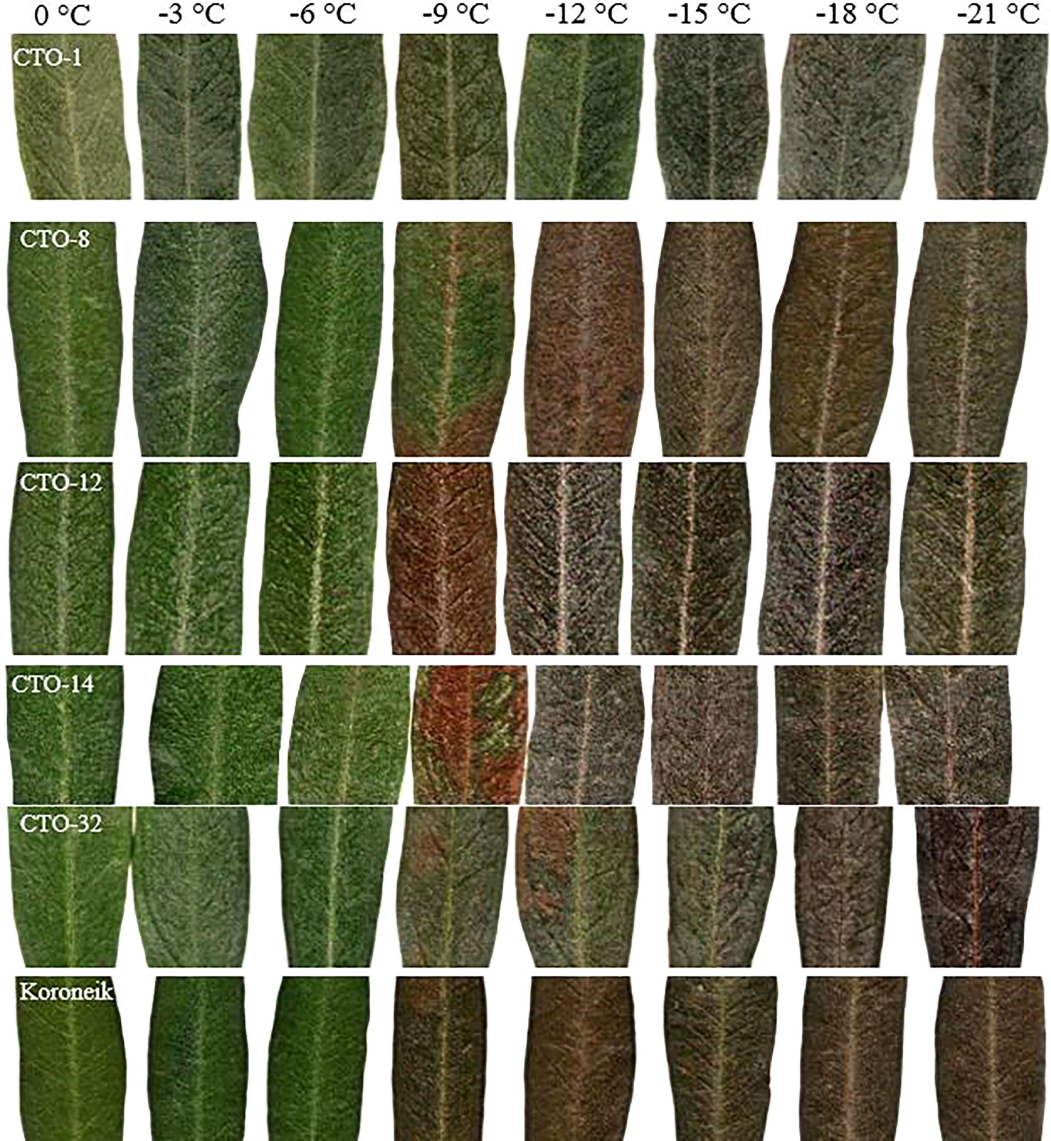
Discoloration of olive leaves at low temperatures in the selected varieties.

To study the effect of artificial cold treatment on the leaves of CTO varieties, five of them with the highest tolerance were selected from the 45 genotypes and were further analyzed. Their leaves were placed in a cold room at different subzero temperatures. The discoloration of olive leaves at low temperatures (from 0°C to −6°C) was not significant, even in the control cultivar (“Koroneiki”). The extent of damage to the leaves differed at temperatures lower than −6°C, depending on the different characteristics of the genotypes (**Figure 7**). The entire leaf area in CTO-12 and “Koroneiki” turned dark at temperatures below −6°C, indicating that they were the most frost-susceptible among the analyzed varieties. Brown and necrotic areas on the upper leaf surface were visible in CTO-8, CTO-14, and CTO-32 at temperatures lower than −9°C, whereas no signs of damage were observed in the leaves of CTO-1 at temperatures lower than −15°C. LT50 values were calculated with a logistic sigmoid function (**Table 5**) and confirmed the observations on the leaf samples. Therefore, genotype CTO-1 was the most tolerant to frost, whereas genotype CTO-12 was the most susceptible genotype and showed symptoms similar to the “Koroneki” cultivar.

**Table 5:** LT50 (°C) estimated by comparing images from the leaves of cold-acclimated varieties.

Varieties	LT_50_
CTO-1	−13.9
CTO-32	−10.8
CTO-08	−9.7
CTO-14	−8.9
CTO-12	−8.1
Koroneiki	−6.32

## 4 Discussion

Iran has valuable genetic reserves of olive trees derived from sexual propagation. Nonetheless, they have not been fully explored in terms of morphological and molecular characteristics. Successful breeding programs can enhance olive yield, quality, and tolerance to environmental stress, although they require a thorough knowledge of the genetic diversity of the available germplasm and an understanding of their ability to cope with climatic changes. Cold tolerance is a complex trait in plants and largely depends on the genetic characteristics of the cultivar, climatic conditions, and management practices in horticulture (Arias et al., 2015). Identifying tolerant varieties after a natural freezing event and comparing them with commercial cultivars are preliminary steps in this direction. Site evaluations of the CTOs showed that some of the trees in the different slopes were similar to each other, such as CTO-11 and CTO-33, which were identified in the northern and western slopes. Although cold tolerance is controlled by genetic factors (Xin and Browse, 2000), environmental cues are associated with physiological and biochemical changes, such as conversion of starch to soluble sugars, thereby causing greater tolerance (Poirier et al., 2010; Arias et al., 2015). In a given species, cold tolerance is a trait that adapts well to the change between summer and winter, but it is also a variable trait that varies from region to region (Bower and Aitken, 2006). Under similar climatic and topographic conditions, the only possible explanation for differences in the symptoms of frost stress in cultivars was reportedly soil type, ground cover, pure water content, and concentration of ice-nucleating bacteria (Snyder and Melo-Abreu, 2005).

The CTO trees showed lower annual vegetative growth, intermediate canopy density, spreading growth habit, and intermediate internode length. In this study, except genetic diversity, the abovementioned morphological traits were relatively similar in almost all CTO olive varieties. Canopy structure can either reduce or increase the probability and severity of freezing in trees (Charrier et al., 2015). This is influenced by the structural arrangement of the foliage, including height, length, density, porosity, and leaf area index (Winkel et al., 2009). The present results do not agree with those of Charrier et al. (2015) who reported that a dense canopy in fruit trees protected susceptible tissues from adverse weather conditions and reduced the probability and severity of frost damage. Contrariwise, but similar to Snyder and Melo-Abreu (2005), our results showed good frost-tolerance in CTO varieties with an intermediate canopy density, light penetration into the canopy, photosynthesis by inner shoots, and accumulation of sugars, compared to a dense canopy. Thus, pruning the dense canopy of trees can increase the entry of light into the canopy and enhance cold tolerance in olive trees.

Significant differences in cold tolerance among olive cultivars were reportedly observed (Barranco et al., 2005; Lodolini et al., 2016; Wang et al., 2018; Saadati et al., 2019). Molecular and morphological analyses of the identified CTO varieties revealed a considerable difference between them, compared to the Iranian and Mediterranean cultivars. The results of the present study showed that many of the CTO trees were genetically closer to the Mediterranean cultivars than the Iranian olive populations. However, Golmohamadi et al. (2018) evaluated 20 Iranian and Mediterranean olive cultivars for cold tolerance and concluded that Iranian cultivars may have a richer gene pool than Mediterranean olives for tolerating cold stress.

To genetically characterize 45 CTO varieties, 10 known SSR markers were used (Baldoni et al., 2009; Mousavi et al., 2017; El Bakkali et al., 2019; Sion et al., 2019; Valeri et al., 2022). The average Ho value was higher than the He value, indicating a high genetic diversity among the CTO germplasm. The dendrogram showed a clear separation of most CTO varieties from other olive varieties and represented a newly discovered reservoir of genetic diversity and cold tolerance in olive trees. Among them, 31 cold-tolerant olive trees were placed in one subcluster (68% of CTO), and nearly half of them (15 CTO) were placed in groups of two or three identical trees. This evidence confirms that some of the varieties had been propagated by vegetative methods, thereby suggesting their possible role in olive oil production in the past. Finally, 36 unique olive varieties were individuated in the present study but were not identical to any of the 321 cultivars analyzed herein. NJ, PCoA, and parentage analyses confirmed the close genetic relationship of four CTO varieties with the Spanish “Picual” cultivar. The cultivars “Frantoio” and “Koroneiki” were susceptible to cold stress (Bartolozzi and Fontanazza, 1999; Gómez-del-Campo and Barranco, 2005; Wang et al., 2018; Reale et al., 2019; Saadati et al., 2019), whereas cultivars “Picual”, “Leccino”, and “Zard” are generally considered cold-tolerant (Barranco et al., 2005; Gómez-del-Campo and Barranco, 2005; Sanzani et al., 2012; Rahemi et al., 2016; Wang et al., 2018; Saadati et al., 2019), thereby confirming our findings. Four unique CTO varieties were clustered in POP2 and were exclusively represented by Iranian varieties. These were strictly related to “Shengeh” and “Zard” cultivars, as further confirmed by NJ analysis. Moreover, three of the four (04, 24 and 38) CTO varieties were unique with a direct parentage of Iranian ecotypes. All other CTO trees that were not assigned to a candidate parent could have originated from seeds and crosses between unknown varieties, along with a major participation of imported Mediterranean cultivars, considering their genetic similarity with this group. These were probably selected, propagated, and maintained by olive growers. Therefore, CTO trees were identified in relatively separate groups from the reference cultivars, thereby increasing the importance of genetic diversity for reaching promising cultivars. In addition, the STRUCTURE analysis, with a K value of 2, allowed the inference of relationships between the CTO varieties. They appeared to have a distinct genetic structure, possibly due to limited exchange or diffusion from the areas of origin. The results of this study agreed well with earlier studies by Mousavi et al. (2014) on the clear separation by cluster and structure analysis in Golestan olive ecotypes and Mediterranean cultivars. They also demonstrated a high differentiation with major Iranian cultivars (Mousavi et al., 2017).

Image comparisons (fractal analysis) are very useful for assessing the cold hardiness of plants based on visible damage (Mancuso et al., 2003; Rallo et al., 2018). The technique is simple, fast, inexpensive, and reliable, compared to other methods (Azzarello et al., 2009). It is based on the degradation of chlorophyll and the release of phenols, indicating a dark color, from damaged cells (Thomashow, 1999; Mancuso et al., 2003; Farooq et al., 2009). Chlorophyll degradation is an indicator of oxidative stress in cold stress caused by phenol oxidation. In the present study, the discoloration of leaves appeared from −9°C in some varieties and spread to lower temperatures in other varieties. Rezaei et al. (2013) studied the resistance of two olive cultivars (Frantoio and Sevillano) to cold stress, showing a high chlorophyll concentration and a lower level of lipid peroxidation in the “Frantoio” cultivar, compared to the “Sevillano” cultivar, meaning less damage from oxidative stress. In such conditions, more phenolic compounds caused the leaves to be darker (Denney et al., 1993).

Finally, the results of the cold tolerance evaluation on cold-adapted leaves clearly showed that at least five CTO varieties are highly resistant to frost. Moreover, the medium-to-high oil content in these varieties demonstrated their agronomic value. Therefore, the evaluation of these varieties for fruit production, oil quantity and quality, tree architecture, and resistance to biotic stress can be a valuable initiative for the establishment of new olive groves. The 36 newly identified local varieties represent a precious gene pool, and the CTOs can be introduced as new olive cultivars or as rootstocks for future breeding programs in the face of climate change scenarios.

## Data availability statement

The original contributions presented in the study are included in the article/**Supplementary Material**, further inquiries can be directed to the corresponding author/s.

## Author contributions

Conceptualizations: MH-M, IK, SN, and KZ; methodology: MH-M, IK, SM, and RM; performed the experiments: IK; analyzed the data: IK, RM, and SM; contributed reagents/materials/analysis software: MH-M and RM; writing—original draft: IK and MH-M; writing—review and editing: MH-M, IK, SM, and RM. All authors contributed to the article and approved the submitted version.
