# Supplementary material for: Cold stress resilience of Iranian olive genetic resources: evidence from autochthonous genotypes diversity
Source: Front Plant Sci. 2023 May 9;14:1140270. doi: 10.3389/fpls.2023.1140270 (PMC10204771; doi:10.3389/fpls.2023.1140270)
Supplement: Supplementary file 1 [file Table_1.docx]

Supplementary Table 1. List of CTO varieties, Iranian ecotypes and Mediterranean varieties

| ***Iranian cold tolerant olive genotypes*** | ***Iranian province of origin*** |
| --- | --- |
| CTO 01 | Golestan |
| CTO 02 | Golestan |
| CTO 03 | Golestan |
| CTO 04 | Golestan |
| CTO 07 | Golestan |
| CTO 08 | Golestan |
| CTO 09 | Golestan |
| CTO 10 | Golestan |
| CTO 11 | Golestan |
| CTO 12 | Golestan |
| CTO 13 | Golestan |
| CTO 14 | Golestan |
| CTO 15 | Golestan |
| CTO 18 | Golestan |
| CTO 19 | Golestan |
| CTO 20 | Golestan |
| CTO 21 | Golestan |
| CTO 22 | Golestan |
| CTO 23 | Golestan |
| CTO 24 | Golestan |
| CTO 28 | Golestan |
| CTO 29 | Golestan |
| CTO 30 | Golestan |
| CTO 32 | Golestan |
| CTO 33 | Golestan |
| CTO 34 | Golestan |
| CTO 35 | Golestan |
| CTO 36 | Golestan |
| CTO 37 | Golestan |
| CTO 38 | Golestan |
| CTO 39 | Golestan |
| CTO 40 | Golestan |
| CTO 41 | Golestan |
| CTO 42 | Golestan |
| CTO 43 | Golestan |
| CTO 44 | Golestan |
| CTO 45 | Golestan |
| CTO 46 | Golestan |
| CTO 47 | Golestan |
| CTO 48 | Golestan |
| CTO 49 | Golestan |
| CTO 50 | Golestan |
| CTO 51 | Golestan |
| CTO 52 | Golestan |
| CTO 53 | Golestan |
| ***Iranian ecotypes*** | ***Iranian province of origi*** |
| Abdanan | Khuzestan |
| Aghili | Khuzestan |
| Avend | Khuzestan |
| Ayeshkalaleh 1 | Golestan |
| Ayeshkalaleh 2 | Golestan |
| Azadshahr 1 | Golestan |
| Azadshahr 2 | Golestan |
| Azadshahr 3 | Golestan |
| Azadshahr 4 | Golestan |
| Banavare 2 | Kermanshah |
| Banavare 3 | Kermanshah |
| Banavare 5 | Kermanshah |
| Banavare 6 | Kermanshah |
| Banavare 7 | Kermanshah |
| Bansarv 1 | Ilam |
| Bansarv 2 | Ilam |
| Bard I | Khuzestan |
| Bekersofla | Fars |
| Biazeh | Esfehan |
| Bibijahan | kohkiluye-boyerahmad |
| Chamjeyeh | Ilam |
| Chamjeyeh Baladast | Ilam |
| Charfarsakh I | kerman |
| Charfarsakh II | kerman |
| Chelisad | Khuzestan |
| Chovar | Ilam |
| Dakal 281 | Khuzestan |
| Dalahoo 1 | Kermanshah |
| Dasht e Deireh 1 | kermanshah |
| Dasht e Deireh 2 | Kermanshah |
| Dastjerd 2 | zanjan |
| Dastjerd 3 | zanjan |
| Dastjerd 4 | zanjan |
| Dehghan | Fars |
| Dehsefid 6 | Kermanshah |
| Dehsefid 8 | Kermanshah |
| Delibayar 1 | kohkiluye-boyerahmad |
| Delibayar 2 | kohkiluye-boyerahmad |
| Delibayar 3 | kohkiluye-boyerahmad |
| Delibayar 4 | kohkiluye-boyerahmad |
| Derak | Fars |
| Dezful 292 | Khuzestan |
| Dezful Safiabad | Khuzestan |
| Dezful Shiraz | Fars |
| Doudakpaeen 1 | Fars |
| Doudakpaeen 2 | Fars |
| Fadak 77 | Ghom |
| Fadak 78 1 | Ghom |
| Fadak 78 5 | Ghom |
| Fadak 86 | Ghom |
| Fath | Ghom |
| Fishomi | fars |
| Fosoon | Khorasan-jenobi |
| Ganaveh | Busher |
| Gardineko | Ilam |
| Ghazanghayeh 1 | Golestan |
| Ghazanghayeh 2 | Golestan |
| Ghazanghayeh 3 | Golestan |
| Ghazanghayeh 4 | Golestan |
| Ghazanghayeh 5 | Golestan |
| Ghazanghayeh 6 | Golestan |
| Ghazanghayeh 7 | Golestan |
| Ghazanghayeh 8 | Golestan |
| Ghazanghayeh 9 | Golestan |
| Gilan e Gharb 1 | Kermanshah |
| Golooleh | NIGEB Greenhouse-Tehran |
| Golooleh II | NIGEB Greenhouse-Tehran |
| Golooleh III | NIGEB Greenhouse-Tehran |
| Golooleh V | NIGEB Greenhouse-Tehran |
| Golooleh VIII | NIGEB Greenhouse-Tehran |
| H1B1 | Bushehr |
| H1B2 | Bushehr |
| H1B3 | Bushehr |
| H1b4 | Bushehr |
| H2b1 | Bushehr |
| H2B2 | Bushehr |
| H2B4 | Bushehr |
| H2B7 | Bushehr |
| Kazerooni | Fars |
| Keveskolya 2 | kohkiluye-boyerahmad |
| Kheirak 2 | Bushehr |
| Kheirak 3 | Bushehr |
| Kheirak 4 | Bushehr |
| Khorma | NIGEB Greenhouse-Tehran |
| Khorma II | NIGEB Greenhouse-Tehran |
| Khorma IV | NIGEB Greenhouse-Tehran |
| Koohlak | charmahal o bakhtiari-shahrekord |
| Koohsaraksonla | kohkiluye-boyerahmad |
| Lemesk | Golestan |
| Livan | Golestan |
| Malekshahi Pashminpaeen | Ilam |
| Mari | NIGEB Greenhouse-Tehran |
| Mavi I | Khuzestan |
| Mondan 1 | kohkiluye-boyerahmad |
| Mondan 2 | kohkiluye-boyerahmad |
| Mondan 3 | kohkiluye-boyerahmad |
| Moshref | Lorestan |
| Nakhchir | Ilam |
| Nasrabad 1 | Golestan |
| Nasrabad 2 | Golestan |
| Ordib | Dasht-e- Kavir |
| Ourmand | charmahal o bakhtiari-shahrekord |
| Parde Ko | Ilam |
| Park e Sarpol 1 | Ilam |
| Park e Sarpol 8 | Ilam |
| Pirdenyar | Ilam |
| Pirhaji 2 | kerman |
| Pirhaji 4 | kerman |
| Pirhaji 6 | kerman |
| Pirhaji 7 | kerman |
| Pirhaji 8 | kerman |
| PoleBreemk5 | kohkiluye-boyerahmad |
| PoleBreemk5 | kohkiluye-boyerahmad |
| Qiup | Khuzestan |
| RG 2 | Roudbar |
| Rowghani | NIGEB Greenhouse-Tehran |
| Rowghani III | NIGEB Greenhouse-Tehran |
| Rowghani V | NIGEB Greenhouse-Tehran |
| Saramad | Lorestan |
| Savary | kohkiluye-boyerahmad |
| Shahdad | Kerman |
| Shapoor I | Fars |
| Shengeh | NIGEB Greenhouse-Tehran |
| Shengeh II | NIGEB Greenhouse-Tehran |
| Shengeh Iii | NIGEB Greenhouse-Tehran |
| Shengeh IV | NIGEB Greenhouse-Tehran |
| Shengeh VI | NIGEB Greenhouse-Tehran |
| Shiraz | Fars |
| Siabdarvish | Ilam |
| Soonak | Charmahal o bakhtiari-shahrekord |
| Tabas | Yazd |
| Tangtamoradi 114493 | kohkiluye-boyerahmad |
| Tangtamoradi K6 | kohkiluye-boyerahmad |
| Torshak | Ilam |
| Uzineh 1 | Golestan |
| Uzineh 2 | Golestan |
| Uzineh 3 | Golestan |
| Varezard | Roudbar |
| Zard | NIGEB Greenhouse-Tehran |
| ***Mediterranean varieties*** | ***Country of origin*** |
| Abbadi | Syria |
| Abouchoki | Syria |
| Aboukanani | Syria |
| Adkam | Syria |
| Agouromanakolia | Greece |
| Alameno de Cabra | Spain |
| Alfafara | Spain |
| Amigdalolia | Greece |
| Arbequina | Spain |
| Arbosana | Spain |
| Ascolana Tenera | Italy |
| Asnal | Spain |
| Ayvalik | Turkey |
| Barri | Syria |
| Beladi | Lebanon |
| Bella di Cerignola | Italy |
| Bentalkadi | Syria |
| Bianchera | Italy |
| Biancolilla | Italy |
| Bical | Spain |
| Blanqueta | Spain |
| Bolvino | Spain |
| Borgiona | Italy |
| Bosana | Italy |
| Boutellain | France |
| Buga | Croatia |
| Caiazzana | Italy |
| Canetera | Spain |
| Canino | Italy |
| Canivano Blanco | Spain |
| Canivano Negro | Spain |
| Capolga | Italy |
| Carbuncion | Italy |
| Carolea | Italy |
| Cassanese | Italy |
| Cellina di Nardo | Italy |
| Chalkidikis | Greece |
| Changlot Real | Spain |
| Chemlal de Kabilye | Algeria |
| Chemlali | Tunisia |
| Cipressino | Italy |
| Cobrancosa | Portugal |
| Coratina | Italy |
| Corbella | Spain |
| Cornezuelo de Jaen | Spain |
| Cornicabra | Spain |
| Crnica | Croatia |
| Cucco | Italy |
| Doebli | Syria |
| Dokkar | Turkey |
| Dolce Agogia | Italy |
| Domat | Turkey |
| Dritta | Italy |
| Elmacik | Turkey |
| Empeltre | Spain |
| Escarabajillo | Spain |
| Farga | Spain |
| Favarol | Italy |
| Frantoio | Italy |
| Galega Vulgar | Portugal |
| Gargna | Italy |
| Gaydoyrelia | Greece |
| Gemlik | Turkey |
| Gentile di Chieti | Italy |
| Gentile di Larino | Italy |
| Gjykatesi | Albania |
| Gordal de Granada | Spain |
| Gordal Sevillana | Spain |
| Grappolo | Italy |
| Grappuda | Italy |
| Grossanne | France |
| Hojiblanca | Spain |
| Imperial | Spain |
| Imperiale | Italy |
| Intosso | Italy |
| Istarska Belica | Croatia |
| Itrana | Italy |
| Izmir Sofralik | Turkey |
| Jabali | Syria |
| Jlot | Syria |
| Joanenca | Spain |
| Kaissy | Syria |
| Kalamon | Greece |
| Kalinjot | Albania |
| Kalokerida | Greece |
| Kan Celebi | Turkey |
| Katodrys | Cyprus |
| Kelbetter | Syria |
| Kerkiras | Greece |
| Khalkhali | Syria |
| Khashabi | Syria |
| Kiraz | Turkey |
| Klirou | Cyprus |
| Klon 14 | Albania |
| Kolybada | Greece |
| Konservolia | Greece |
| Koroneiki | Greece |
| Kotruvsi | Albania |
| Lastovka | Croatia |
| Leccino | Italy |
| Lechin de Granada | Spain |
| Lechin de Sevilla | Spain |
| Levantinka | Croatia |
| Llumeta | Spain |
| Lucques | France |
| Maarri | Syria |
| Machorron | Spain |
| Madural | Portugal |
| Mahati | Syria |
| Manzanilla Cacerena | Spain |
| Manzanilla de Almeria | Spain |
| Manzanilla de Sevilla | Spain |
| Marsaline | Tunisia |
| Masabi | Syria |
| Mastoidis | Greece |
| Maureya | Greece |
| Maurino | Italy |
| Mawi | Syria |
| Memecik | Turkey |
| Meski | Tunisia |
| Mignola | Italy |
| Mirtolia | Greece |
| Mollar de Cieza | Spain |
| Moraiolo | Italy |
| Morisca | Spain |
| Negrillo Redondo | Spain |
| Nocellara del Belice | Spain |
| Nostrale di Rigali | Italy |
| Nostrana di Brisighella | Italy |
| Oblica | Croatia |
| Ocal | Spain |
| Ogliarola Salentina | Italy |
| Olivago | Italy |
| Oliviere | France |
| Orbetana | Italy |
| Ottobratica | Italy |
| Ouslati | Tunisia |
| Palomar | Spain |
| Passalunara | Italy |
| Pendolino | Italy |
| Piantone di Mogliano | Italy |
| Picholine | France |
| Picholine Marocaine | Morocco |
| Picolimon | Spain |
| Picual | Spain |
| Picudo | Spain |
| Pignola | Italy |
| Pisciottana | Italy |
| Racioppa | Italy |
| Raio | Italy |
| Rapasayo | Spain |
| Ravece | Italy |
| Rosciola | Italy |
| Royal de Cazorla | Spain |
| Safrawi | Syria |
| Salonenque | France |
| Salvia | Italy |
| Sant Agostino | Italy |
| Sayfi | Syria |
| Semidana | Italy |
| Sevillenca | Spain |
| Shami | Spain |
| Sirole | Italy |
| Sorani | Syria |
| Sourani Red | Syria |
| Tanche | France |
| Toffahi | Egypt |
| Trylia | Syria |
| Ulliri I Bardhe I Tiranes | Albania |
| Ulliriikuq | Albania |
| Uovo di Piccione | Italy |
| Uslu | Turkey |
| Valanolia | Greece |
| Verdale | France |
| Verdial de Badajoz | Spain |
| Verdial de Huevar | Spain |
| Villalonga | Spain |
| Wardan | Egypt |
| Yun Celebi | Turkey |
| Zaituna | Italy |
| Zaity | Syria |
| Zalmati | Tunisia |
