# Supplementary material for: Cold stress resilience of Iranian olive genetic resources: evidence from autochthonous genotypes diversity
Source: Front Plant Sci. 2023 May 9;14:1140270. doi: 10.3389/fpls.2023.1140270 (PMC10204771; doi:10.3389/fpls.2023.1140270)
Supplement: Supplementary file 2 [file Table_2.docx]

Supplementary Table 2. Fruit weight, fruits moisture and oil content of dry weight of the selected CTO varieties.

| Variety | Fruit weight (g) | Fruit moisture (%) | Dried Fruit oil content (%) |
| --- | --- | --- | --- |
| CTO-1 | 1.18 | 69.43 | 34.74 |
| CTO-2 | 2.78 | 45.60 | 43.35 |
| CTO-3 | 2.30 | 55.34 | 47.35 |
| CTO-8 | 1.20 | 46.41 | 35.48 |
| CTO-11 | 1.61 | 42.29 | 35.42 |
| CTO-12 | 1.22 | 46.24 | 35.96 |
| CTO-13 | 1.58 | 44.17 | 40.20 |
| CTO-14 | 0.91 | 39.33 | 41.17 |
| CTO-15 | 1.97 | 46.89 | 45.06 |
| CTO-19 | 1.77 | 52.03 | 47.72 |
| CTO-20 | 2.64 | 47.41 | 44.01 |
| CTO-21 | 1.07 | 41.21 | 16.59 |
| CTO-28 | 2.38 | 43.60 | 39.95 |
| CTO-29 | 1.61 | 37.11 | 32.79 |
| CTO-30 | 1.09 | 40.44 | 42.92 |
| CTO-32 | 1.21 | 44.17 | 30.74 |
| CTO-34 | 2.09 | 49.27 | 45.24 |
| CTO-35 | 2.62 | 48.53 | 44.26 |
| CTO-40 | 2.34 | 53.57 | 46.12 |
| CTO-41 | 2.01 | 51.80 | 49.91 |
| CTO-49 | 0.51 | 35.44 | 14.95 |
| CTO-50 | 2.07 | 45.60 | 47.54 |
| CTO-51 | 2.10 | 50.59 | 44.03 |
| CTO-53 | 1.55 | 57.36 | 43.81 |
